# Post-Exercise Muscle Protein Synthesis in Rats after Ingestion of Acidified Bovine Milk Compared with Skim Milk

**DOI:** 10.3390/nu9101071

**Published:** 2017-09-27

**Authors:** Kyosuke Nakayama, Atsushi Kanda, Ryoichi Tagawa, Chiaki Sanbongi, Shuji Ikegami, Hiroyuki Itoh

**Affiliations:** Food Science Research Labs., Meiji Co., Ltd., 540 Naruda, Odawara, Kanagawa 250-0862, Japan; atsushi.kanda.ba@meiji.com (A.K.); ryouichi.tagawa@meiji.com (R.T.); chiaki.sanbongi@meiji.com (C.S.); shuuji.ikegami@meiji.com (S.I.); hiroyuki.itou@meiji.com (H.I.)

**Keywords:** milk protein, leucine, FSR, mTOR signaling, hyperaminoacidemia

## Abstract

Bovine milk proteins have a low absorption rate due to gastric acid-induced coagulation. Acidified milk remains liquid under acidic conditions; therefore, the absorption rate of its protein may differ from that of untreated milk. To investigate how this would affect muscle protein synthesis (MPS), we compared MPS after ingestion of acidified versus skim milk in rats. Male Sprague-Dawley rats swam for 2 h and were immediately administered acidified or skim milk, then euthanized at 30, 60, 90, and 120 min afterwards. Triceps muscle samples were excised for assessing fractional synthetic rate (FSR), plasma components, intramuscular free amino acids and mTOR signaling. The FSR in the acidified milk group was significantly higher than in the skim milk group throughout the post-ingestive period. Plasma essential amino acids, leucine, and insulin levels were significantly increased in the acidified milk group at 30 min after administration compared to the skim milk group. In addition, acidified milk ingestion was associated with greater phosphorylation of protein kinase B (Akt) and ribosomal protein S6 kinase (S6K1), and sustained phosphorylation of 4E-binding protein 1 (4E-BP1). These results indicate that compared with untreated milk, acidified milk ingestion is associated with greater stimulation of post-exercise MPS.

## 1. Introduction

Skeletal muscle is the most common of the three types of muscle (skeletal muscle, smooth muscle, and cardiac muscle), and it is important to increase or maintain skeletal muscle mass to improve or maintain muscle performance, especially in athletes. The amount of skeletal muscle mass is regulated by the balance between muscle protein synthesis (MPS) and breakdown [1]. Skeletal muscle hypertrophy requires that MPS must exceed breakdown, and accelerating MPS is more important to enhance the muscle protein anabolic response [2,3,4]. Exercise is well known to increase the rate of post-exercise MPS [3,5]. Consumption of amino acids or protein-rich foods also stimulates MPS [6,7]; in fact, exercise and amino acid intake can have additive effects on MPS [8,9].

Bovine milk proteins are of the highest quality because they possess a complete profile of essential amino acids [10], have high amino acid digestibility and absorptivity [11,12], and are associated with marked changes in MPS [13,14] through the activation of signaling of the mammalian target of rapamycin (mTOR) [15]. Furthermore, milk has the potential to be a good protein supplement over the course of a long training period [16]. However, bovine milk, including skim milk, coagulates in acidic conditions (e.g., gastric acid) due to the property of casein, the most common of the bovine milk proteins [17]. Pectin and soybean polysaccharide have commonly been used as stabilizers in acidified milk dispersions [18], such as yogurt beverages. We designed and developed acidified milk using a unique procedure involving addition of stabilizers, as well as a dispersant, namely fermented cellulose. The acidification of the milk with the stabilizers and fermented cellulose can suppress the aggregation of milk proteins so that the milk can maintain liquidity with low viscosity under acidic conditions while containing the same or a higher amount of protein compared with untreated bovine milk.

Due to the stability of acidified milk under acidic conditions, acidified milk is less likely to coagulate in the stomach, compared with bovine milk. Therefore, the absorption rate of its proteins may differ from those of bovine milk. The differences of absorption rate among dietary proteins appear to affect postprandial protein synthesis as has been seen in whey protein, which is characterized as “fast” protein, compared with casein as “slow” protein [19,20,21,22,23]. We, therefore, hypothesized that MPS stimulation following ingestion of acidified milk would differ from that with bovine milk because even though they contain the same types of proteins, the protein absorbability may differ. In the present study, we measured protein synthesis in muscle tissue from rats that ingested acidified milk compared with skim milk after exercise. We also compared mTOR signaling in muscle tissue from the two groups. We also compared these parameters in the two groups versus an exercise-only control group that was administered neither of the milk solutions.

## 2. Materials and Methods

### 2.1. Experimental Animal Protocol

Male Sprague-Dawley rats with body weights of approximately 150 g (CLEA Japan, Inc., Tokyo, Japan, *n* = 68) were used in this study. The animals were maintained at 22 ± 2 °C, with lights on from 7:00 am until 7:00 pm, and had free access to water and food (protein 23.6%, fat 5.3%, carbohydrate 54.4%, ash 6.1%, fiber 2.9% and moisture 7.7% (MF; Oriental Yeast Company Ltd., Tokyo, Japan). This study was approved by the Animal Committee of the Food Science Research Laboratory, Meiji Company Limited (Kanagawa, Japan), with animals receiving care according to the guidelines laid down by this committee (protocol nos. 2014_3871_0142, 0143, and 0144).

**Figure 1:**
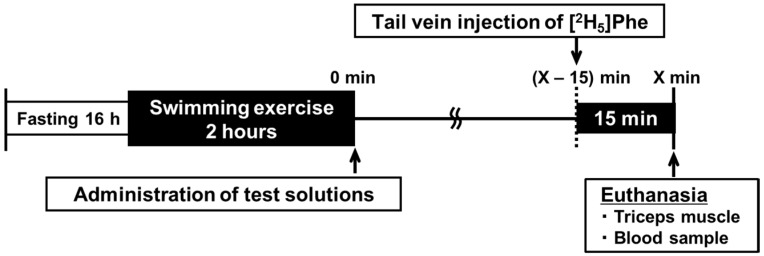
The experimental protocol. Acidified milk and skim milk groups: *X* = 30, 60, 90, or 120. Exercise-only control group (without administration of either test solution): *X* = 15.

The experimental protocol is shown in Figure 1. The swimming exercise protocol matched a protocol previously described [15]. The rats were acclimated to swimming for 30 min by training that began 2 days before the experiment. For the 16 h prior to the start of the experiment, the rats were fasted. On the day of the experiment, the rats swam for 2 h, with four rats swimming simultaneously in a barrel filled to a depth of 50 cm, yielding an average surface area of 400 cm^2^ for each rat. Water temperature was maintained at 34 °C during the swimming protocol. Immediately following exercise, the rats were divided into two groups. Each group was administered one of two isonitrogenous test solutions in oral form using a sonde (2.4 mL/100 g body weight (BW), 0.85 g protein/kg BW). At 30, 60, 90, and 120 min after the administration, rats in both groups (*n* = 6–8/group/time point) were anesthetized with 30% isoflurane diluted with propylene glycol and then euthanized by exsanguination. A control group of rats (*n* = 8) were similarly anesthetized and euthanized 15 min after exercise but without administration of either test solution beforehand (the exercise-only control group). Blood samples were taken from the inferior vena cava, and plasma was isolated from the blood samples. Triceps muscle were excised and stored at −80 °C until analysis.

### 2.2. Administration of Metabolic Tracer

At 15 min before euthanization, a bolus dose (45 mg/kg BW, 22.5 mg/mL) of ^2^H-labeled phenylalanine ([^2^H_5_]Phe; Cambridge Isotope Laboratories, Inc., Tewksbury, MA, USA) was injected via the tail vein for measurement of the protein fractional synthesis rate (FSR). At 15 min after the injection, the triceps muscle was excised and frozen rapidly. The elapsed time from injection until freezing was recorded as the actual time for incorporation of the labelled amino acid into the protein.

### 2.3. Test Solutions

We used two isonitrogenous test solutions: acidified milk and skim milk. Table 1 shows the nutritional composition of each solution. Acidified milk was prepared by following the steps below:

(1) Solution A: 433 g of milk protein concentrate (Idaho Milk Products, Inc., Jerome, ID, USA) were dissolved in 4067 g of water by heating.
(2) Solution B: 40 g of soybean polysaccharide (San-Ei Gen F.F.I., Inc., Tokyo, Japan), 10 g of pectin (UNITEC FOODS Co., Ltd., Tokyo, Japan), 5 g of fermented cellulose (San-Ei Gen F.F.I., Inc., Tokyo, Japan), 100 g of trehalose, and 340 g of high-fructose corn syrup were dissolved in 4005 g of water.
(3) Solution C: 63 g of citric acid (SODA AROMATIC Co., Ltd., Tokyo, Japan) were dissolved in 937 g of water.
(4) Solution A and B were mixed and solution C was gradually added to the mixture. The resulting mixture was homogenized and then used as the test solution. The pH of acidified milk was 4.1 and the viscosity of acidified milk was 10 mPa·s at 10 °C.

Skim milk was prepared by dissolving 97 g of skim milk powder (Meiji Co., Ltd., Tokyo, Japan) in 903 g of water. The pH of skim milk was 6.9.

**Figure 2:**
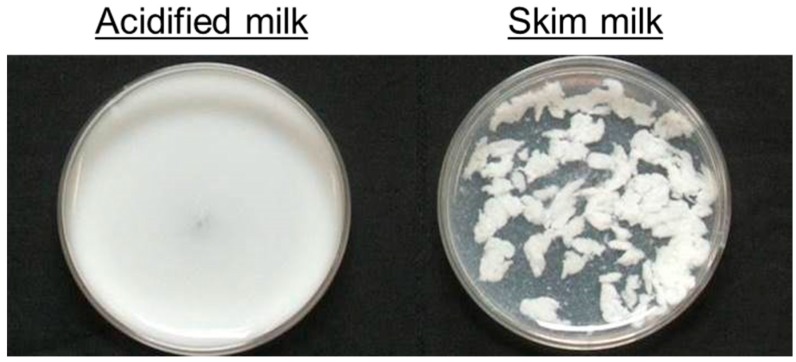
A photographic image of the acidified milk (**left**) and skim milk (**right**) mixed with artificial gastric juice.

Figure 2 shows a photographic image of the acidified milk and the skim milk, each mixed with artificial gastric juice (0.6% (*w/v*) pepsin in the first fluid for disintegration test, pH 1.2, Kanto Chemical Co., Inc., Tokyo, Japan). The acidified milk remained as a solution, but clots formed in the skim milk.

### 2.4. Plasma Measurements

Plasma insulin levels were measured using a commercial ELISA kit for rat insulin (Mercodia Rat Insulin ELISA, Mercodia AB, Uppsala, Sweden), and plasma amino acids were measured by high-performance liquid chromatography with pre-column 6-aminoquinolyl-*N*-hydroxysuccinimidyl carbamate derivatization [24].

### 2.5. Measurement of Muscle Protein Synthesis (MPS)

The rate of MPS was determined by measuring the incorporation of the injected [^2^H_5_]Phe into triceps muscle proteins, using a procedure described previously [25]. Briefly, frozen triceps muscle samples (565–785 mg) were weighed and homogenized in ice-cold 3% ((*w/v*)) perchloric acid. After centrifugation, the supernatants were collected and the pellets were further washed with distilled water and hydrolyzed with hydrochloric acid. The enrichment of [^2^H_5_]Phe in intramuscular free amino acids was measured in the supernatants and the muscle protein-bound [^2^H_5_]Phe enrichment was measured in the hydrolyzed muscle protein pellets using liquid chromatography tandem-mass spectrometry (ACQUITY TQD, Waters Corporation, Milford, MA, USA). We calculated the FSR, defined as the percentage of muscle protein renewed each day, of triceps muscle proteins according to the formula:

FSR = (Eb × 100)/(Ea × t)
(1)

where Eb is the protein-bound Phe enrichment, Ea is the Phe enrichment in the free intramuscular pool, and t is the time interval between the injection of the tracer and the cooling of the muscle sample, expressed in days.

### 2.6. Intramuscular Free Amino Acid Concentrations

The supernatants of perchloric acid extracts of triceps muscle tissues were assayed for intramuscular free amino acids by high-performance liquid chromatography with pre-column 6-aminoquinolyl-*N*-hydroxysuccinimidyl carbamate derivatization [24].

### 2.7. Western Blot Analysis

Western blot analysis was performed using a modification of a procedure described by Kido et al. [26]. Muscle samples (100 mg) were homogenized in ice-cold RIPA buffer (Cell Signaling Technology, Danvers, MA, USA) with a protease and phosphatase inhibitor cocktail (PhosSTOP and Complete Mini EDTA-free, Sigma-Aldrich Co., Ltd., St. Louis, MO, USA). The homogenates were centrifuged at 14,000× g for 20 min at 4 °C. The total protein concentration of the tissue homogenate supernatants was measured using the bicinchoninic acid assay (TaKaRa BCA Protein Assay Kit, Takara Bio Inc., Shiga, Japan) with bovine serum albumin (Takara Bio Inc., Shiga, Japan) as the standard. The samples were diluted in 3× sample buffer (15.0% (*v/v*) β-mercaptoethanol, 9.0% (*w/v*) sodium dodecyl sulfate, 0.195 mol/L Tris-HCl, pH 6.8, 30% (*v/v*) glycerol, and 0.0075% (*w/v*) bromophenol blue (all reagents were purchased from Wako Pure Chemical Industries, Ltd., Osaka, Japan) and incubated at 95 °C for 10 min. Using 5–20% SDS-polyacrylamide gels (Perfect NT Gel, DRC, Co., Ltd., Tokyo, Japan), 20-μg protein samples were separated by electrophoresis and subsequently transferred to polyvinylidene difluoride membranes (Immobilon-P, Merck KGaA, Darmstadt, Germany). After the transfer, the membranes were washed in Tris-buffered saline containing 0.1% Tween 20 (TBST), and then blocked with 5% (*w/v*) skim milk powder in TBST for 1 h at room temperature. After blocking, the membranes were washed and incubated overnight at 4 °C with the primary antibodies p-Akt (Ser473), total Akt, p-S6K1 (Thr389), total S6K1, p-4E-BP1 (Thr37/46), and total 4E-BP1 (Cell Signaling Technology, Danvers, MA, USA). The membranes were then washed again in TBST and incubated for 1 h at room temperature with a secondary antibody (Anti-rabbit IgG, HRP-linked Antibody, Cell Signaling Technology, Danvers, MA, USA). Chemiluminescent reagents (Luminata Forte Western HRP Substrate, Merck KGaA, Darmstadt, Germany) were used to facilitate the detection of protein bands, which were then visualized using the ChemiDoc XRS system (Bio-Rad Laboratories, Inc., Hercules, CA, USA). Phosphorylation levels were determined by expression of phosphorylated protein divided by expression of nonphosphorylated total protein.

### 2.8. Statistical Analysis

All values are expressed as mean ± standard error of the mean (SEM). Since the exercise-only control group received no treatment, the data were first analyzed using a one-factor (1 × 9) analysis of variance (ANOVA) with a Dunnett’s post hoc test for comparison of the treatment group with the exercise-only control group. The data were then analyzed using a two-factor (2 × 4; treatment, time) ANOVA. Differences among individual means were assessed by using Tukey’s post hoc test when significant interactions between treatment and time were found. All analyses were performed by using SPSS for Windows, version 23 (IBM Japan, Ltd., Tokyo, Japan). Significance was set at *p* < 0.05.

## 3. Results

### 3.1. Fractional Synthetic Rate (FSR)

**Figure 3:**
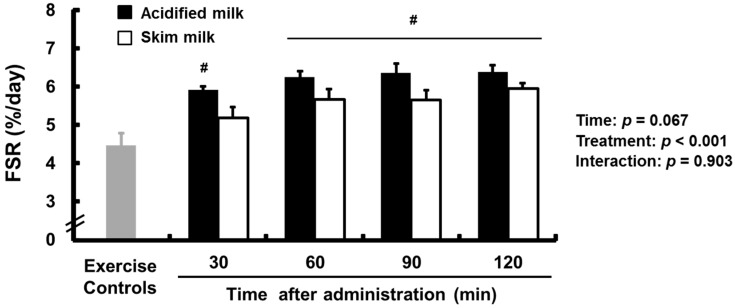
The FSR of triceps muscle tissue from rats administered acidified milk or skim milk. Data are presented as means ± SEM (*n* = 6–8). ^#^ Significantly different from the exercise-only control group, *p* < 0.05. FSR, fractional synthetic rate; SEM, standard error of the mean.

Triceps muscle protein FSR data are presented in Figure 3. Compared with the exercise-only control group, the mean FSR was significantly higher (*p* < 0.05) in the acidified milk group at each time point and in the skim milk group at 60, 90, and 120 min after administration. The mean FSR in the acidified milk group was significantly higher than that of the skim milk group (main effect for treatment: *p* < 0.001; treatment × time: *p* = 0.903).

### 3.2. Plasma Amino Acids and Insulin 

**Figure 4:**
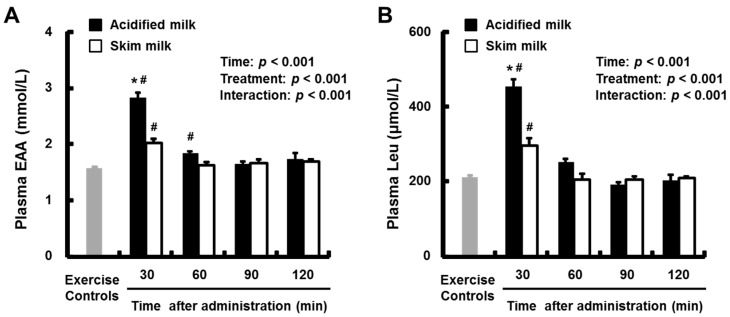
Plasma EAA (**A**) and Leu (**B**) levels in rats administered acidified milk or skim milk. Data are presented as means ± SEM (*n* = 6–8). * Significantly different from the skim milk group at the same time point. ^#^ Significantly different from the exercise-only control group, *p* < 0.05. EAA, essential amino acids; Leu, leucine; SEM, standard error of the mean.

Plasma essential amino acids (EAA) and leucine (Leu) levels are presented in Figure 4A,B. The mean plasma EAA and Leu levels in both treatment groups at 30 min after administration were higher than in the exercise-only control group, and the mean plasma EAA levels in the acidified milk group at 60 min were also higher than in the exercise-only control group (*p* < 0.05). Significant treatment × time interactions (*p* < 0.001) were found in the mean plasma EAA and Leu levels. The acidified milk group had significantly higher (*p* < 0.05) mean plasma EAA and Leu levels compared with the skim milk group at 30 min.

**Figure 5:**
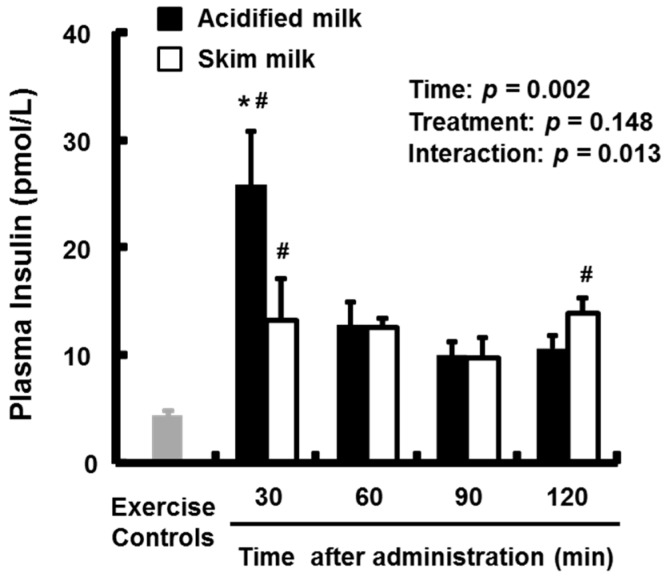
Plasma insulin levels in rats administered acidified milk or skim milk. Data are presented as means ± SEM (*n* = 6–8). * Significantly different from skim milk group at same time point. ^#^ Significantly different from exercise-only controls, *p* < 0.05. SEM, standard error of the mean.

Plasma insulin levels are presented in Figure 5. The mean plasma insulin levels in both groups at 30 min and in the skim milk group at 120 min were higher than in the exercise-only group (*p* < 0.05 for all between-group differences). Significant treatment × time interactions (*p* = 0.013) were found in the plasma insulin levels. The acidified milk group had a significantly higher (*p* < 0.05) mean plasma insulin level compared with the skim milk group at 30 min.

### 3.3. Intramuscular Free Amino Acids

**Figure 6:**
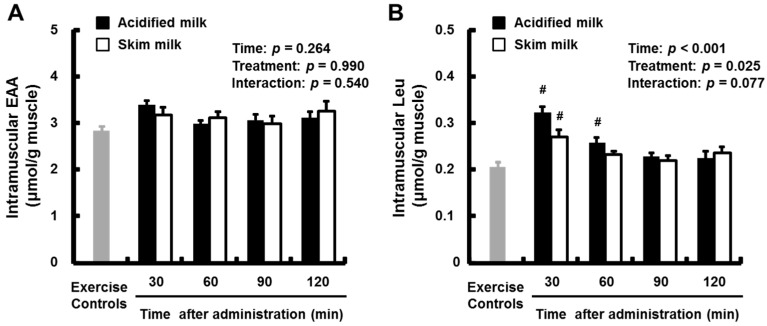
Intramuscular free EAA (**A**) and Leu (**B**) levels in rats administered acidified milk or skim milk. Data are presented as means ± SEM (*n* = 6–8). ^#^ Significantly different from exercise-only controls, *p* < 0.05. EAA, essential amino acids; Leu, leucine; SEM, standard error of the mean.

Intramuscular free EAA and Leu levels are presented in Figure 6A,B, respectively. There were no significant changes in the mean intramuscular EAA levels over time or when comparing the milk groups with each other or with the exercise-only control group. The mean intramuscular Leu levels in both milk groups at 30 min after administration were higher than in the exercise-only control group, and the mean level in the acidified milk group at 60 min was also higher than in the exercise-only control group (*p* < 0.05). The mean intramuscular Leu level in the acidified milk group was significantly higher than in the skim milk group (main effect for treatment: *p* = 0.025; treatment × time: *p* = 0.077).

### 3.4. Phosphorylated Akt, S6K1, and 4E-BP1 Levels

**Figure 7:**
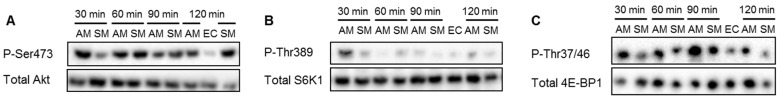
Western blotting images of Akt Ser473 (**A**); S6K1 Thr389 (**B**); and 4E-BP1 Thr37/46 (**C**) in rats administered acidified milk or skim milk. AM, acidified milk; SM, skim milk; EC, exercise-only controls.

Phosphorylated Akt (Ser473), S6K1 (Thr389), and 4E-BP1 (Thr37/46) levels are presented in Table 2, and the Western blotting images are presented in Figure 7A–C. Significant treatment × time interactions (*p* < 0.05) were found in the mean levels of phosphorylated Akt and S6K1, but there were no significant interactions for the mean levels of phosphorylated 4E-BP1. Compared with the exercise-only group, phosphorylated Akt levels were significantly higher at each time point and phosphorylated 4E-BP1 levels were significantly higher at 60 min after administration in both of the milk groups (*p* < 0.05). Levels of phosphorylated S6K1 at 30 min and 4E-BP1 at 90 min significantly increased only in the acidified milk group (*p* < 0.05). The mean levels of phosphorylated Akt and S6K1 at 30 min in the acidified milk group were significantly higher (*p* < 0.05) than in the skim milk group.

## 4. Discussion

In the present study, we developed a unique preparation of acidified milk, and we found that this preparation was associated with greater increases in post-exercise MPS compared with skim milk in spite of containing the same types of protein as skim milk. To our knowledge, this is the first study that demonstrated that processing a milk protein solution created differences in its physiological properties.

Acidified milk, such as in yogurt beverages, is commonly prepared by the coagulation of dairy milk under acidic conditions, followed by dilution, homogenization, and the addition of stabilizers. The acidic milk we developed was prepared by a unique procedure in which milk protein solution, stabilizers and a dispersant were mixed in advance, and then acid was gradually added to the mixture. The acidified milk contained a high amount of milk protein, equal to or greater than that in bovine milk, and maintained liquidity with low viscosity under acidic conditions. In the present study, we found that ingestion of the acidified milk was associated with significantly greater post-exercise MPS compared with skim milk ingestion in rats.

Interestingly, both the acidified milk and skim milk had the same types and quantities of milk protein. While previous studies have shown that amino acid composition [27,28] and the amount of protein intake [15,29,30] were important factors in determining the effects on MPS, the present study suggested that “aminoacidemia” affected the muscle protein anabolic response. Ingestion of the acidified milk was associated with an acute increase in plasma EAA, including leucine. It is well known that EAA, especially leucine, play an important role in the activation of MPS [27,31,32]. Pennings et al., observed a strong positive correlation between peak plasma leucine concentrations and postprandial FSR values [21]. The increased levels of plasma EAA, especially leucine, in the acidified milk group would probably have caused greater stimulation of MPS compared to the skim milk group. Since the acidified milk maintained its liquidity under acidic conditions, its proteins may have transited more rapidly from the stomach to the small intestine and have been absorbed faster than those of skim milk. This difference in transit time might have caused the acute response of plasma EAA and leucine, as is the case with whey protein. The gastric emptying rate of whey protein is higher and is associated with faster and greater aminoacidemia than casein, because whey protein is a soluble protein in the stomach whereas casein is not [17,19]. Holwerda et al., showed that delay of gastric emptying was accompanied by a lesser postprandial increase in plasma amino acid concentrations following protein ingestion [33]. Consequently, it is important to increase the gastric emptying rate of protein for inducing hyperaminoacidemia, and that would be applicable to the acidified milk in the present study. However, we have not measured the gastric emptying and absorption rate of proteins directly, so further study is necessary to confirm the speculation.

The relationships between postprandial aminoacidemia and MPS have been investigated through the comparison of whey protein and casein, which have complete profiles of EAA and high absolute absorbability but differing the rate of absorption [19]. Among the studies in humans, several studies [21,23,34] showed that whey protein, which caused hyperaminoacidemia because of its high absorption rate, was associated with greater activation of MPS than casein, which caused lower aminoacidemia than whey protein, while other studies [20,22] showed that the proteins were associated with similar levels of MPS stimulation. In our previous animal study [15], ingestion of whey protein after exercise was associated with increases in MPS at an early time point (by 60 min after ingestion), while casein was associated with increases in MPS at a later time point (by 120 min after ingestion), and the peak values of FSR in both groups were similar. In the present study, ingestion of acidified milk, which caused hyperaminoacidemia, was associated with a greater increase of MPS throughout the post-ingestion period (30–120 min after ingestion) compared with skim milk, which caused lower aminoacidemia than acidified milk. The discrepancy between the MPS results in the two studies might have been due to the difference in the doses of protein. The dose in our previous study was 3.1 g protein/kg BW, and that of the present study was 0.85 g protein/kg BW, because we used test solutions in the present study that contained concentrations of protein that were similar to those of raw bovine milk. The dose of protein in the previous study [15] was associated with maximal stimulation of MPS so that not only whey protein but also casein-induced substantial elevation of plasma EAA for stimulating MPS. By contrast, the dose in the present study was associated with submaximal stimulation of MPS, and the aminoacidemia following skim milk ingestion might have been insufficient to stimulate MPS strongly unlike the acidified milk. In two studies of human subjects that had indicated the predominance of whey protein in stimulating MPS compared to casein [21,23], the doses of protein were associated with submaximal stimulation of MPS. This was because the studies had elderly individuals as the subjects and used approximately 20 g of protein as the dose, although the dose of protein needed for maximal enhancement of MPS in the elderly individuals appeared to be 40 g [7,35]. On the contrary, the studies that concluded that whey protein and casein similarly enhanced MPS used young men as subjects and used doses previously associated with maximal MPS stimulation in young men (approximately 20 g) [29,30]. We speculated that inducing hyperaminoacidemia can cause a greater increase of MPS even under submaximal protein intake conditions.

We analyzed the phosphorylation status of muscle anabolic signaling proteins to support the results of FSR. It is well known that mTOR is an important modulator of MPS [36]. Ribosomal protein S6 kinase (S6K1) is one of the downstream targets of mTOR, and previous studies have indicated a positive correlation between the acute phosphorylation of S6K1 and long-term increases in muscle mass in rodents [37] and humans [38]. In this study, higher levels of phosphorylated S6K1 were observed at 30 min after administration of acidified milk compared to skim milk, which may be related to enhanced MPS in the acidified milk group. Increasing phosphorylation of S6K1 in the acidified milk group may have been caused by high plasma and intramuscular levels of leucine, because amino acids, particularly leucine, can activate mTOR [39,40]. mTOR is also positively regulated by protein kinase B (Akt) directly [41] or indirectly [42]. Phosphorylation of Akt in the acidified milk group was significantly higher than in the skim milk group at 30 min after administration, which may have been caused by higher plasma insulin levels following acidified milk administration compared to skim milk. The increased Akt phosphorylation might have led to more phosphorylation of S6K1 via mTOR activity. Unlike the acute response of S6K1, the increase in phosphorylation of 4E-binding protein 1 (4E-BP1), which is also one of the downstream targets of mTOR, was relatively moderate and reached a peak at a later time point. Previous studies have indicated the activation of both S6K1 and 4E-BP1 with similar pattern [32,43,44], but several studies have reported the discrepancy of phosphorylation between S6K1 and 4E-BP1 with amino acids or protein supplementation [22,45,46]. The longer time of activation of 4E-BP1 in the acidified milk group compared to the skim milk group could have caused the enhancement of MPS during the later time period in the study.

Several limitations exist in this study. First, we have not measured the gastric emptying and absorption rate of proteins directly, so it is possible that factors other than the absorption rate of proteins were associated with the differences in postprandial aminoacidemia and MPS observed in rats administered the acidified milk compared with the skim milk. Second, this is an animal study, and the results might not necessarily be applicable to humans. Studies of human subjects are necessary to further verify the effects of ingestion of the acidified milk.

## 5. Conclusions

In conclusion, the present results demonstrate that ingestion of the newly-designed acidified milk was associated with greater post-exercise MPS compared with skim milk through greater increases of plasma EAA, leucine and insulin levels, and increased mTOR signaling as shown by increased phosphorylation of its downstream targets. These results indicate that inducing hyperaminoacidemia might be one of the effective strategies to enhance MPS under submaximal protein intake conditions. Although further studies analyzing human subjects are needed, it is an important finding for sports nutrition that consumption of a processed milk protein solution may improve postprandial muscle metabolism following exercise.

## Figures and Tables

**Table 1:** The nutritional composition of the test solutions.

Component	Acidified Milk	Skim Milk
Energy (kcal/100 g)	32	33
Protein (g/100 g)	3.4	3.4
Carbohydrate (g/100 g)	4.5	4.7
Fat (g/100 g)	0.1	0.1

**Table 2:** Western blotting analyses of synthesis-associated signaling proteins in rats administered acidified milk or skim milk ^1^.

	Time after Administration				*p* (ANOVA)		
	30 min	60 min	90 min	120 min	Time	Treatment	Interaction
	Phosphorylated/total, fold of exercise-only controls						
Akt Ser473							
Acidified milk	4.32 ± 0.42 *^,#^	3.00 ± 0.47 ^#^	2.37 ± 0.30 ^#^	2.81 ± 0.21 ^#^	0.149	0.393	0.010
Skim mik	2.71 ± 0.31 ^#^	2.89 ± 0.41 ^#^	3.14 ± 0.35 ^#^	2.93 ± 0.19 ^#^			
S6K1 Thr389							
Acidified milk	3.55 ± 0.99 *^,#^	1.26 ± 0.13	1.59 ± 0.16	1.41 ± 0.21	0.002	0.014	0.006
Skim mik	1.47 ± 0.18	1.43 ± 0.14	1.13 ± 0.13	1.44 ± 0.10			
4E-BP1 Thr37/46							
Acidified milk	2.35 ± 0.26	2.89 ± 0.59 ^#^	2.64 ± 0.37 ^#^	2.20 ± 0.28	0.148	0.179	0.677
Skim mik	1.93 ± 0.30	3.01 ± 0.81 ^#^	1.67 ± 0.41	1.76 ± 0.34			

^1^ Values are mean ± SEM. * Significantly different from the skim milk group at the same time point, *p* < 0.05. ^#^ Significantly different from the exercise-only controls, *p* < 0.05. ANOVA, analysis of variance; SEM, standard error of the mean.
